# Treatment of high-strength ethylene glycol waste water in an expanded granular sludge blanket reactor: use of PVA-gel beads as a biocarrier

**DOI:** 10.1186/s40064-016-2409-9

**Published:** 2016-06-23

**Authors:** Yue Jin, Dunqiu Wang, Wenjie Zhang

**Affiliations:** Guangxi Key Laboratory of New Energy and Building Energy Saving, College of Civil Engineering and Architecture, Guilin University of Technology, 12, Jiangan Road, Guilin, 541004 China; Guangxi Key Laboratory of Environmental Pollution Control Theory and Technology, Guilin University of Technology, Guilin, 541004 China; Guangxi Collaborative Innovation Center for Water Pollution Control and Water Safety in Karst Area, Guilin University of Technology, Guilin, 541004 China

**Keywords:** Anaerobic, Cost, *Methanosaeta*, *Methanosarcina*

## Abstract

Industrial-scale use of polyvinyl alcohol (PVA)-gel beads as biocarriers is still not being implemented due to the lack of understanding regarding the optimal operational parameters. In this study, the parameters for organic loading rate (OLR), alkalinity, recycle rate, and addition of trace elements were investigated in an expanded granular sludge blanket reactor (EGSB) treating high-strength ethylene glycol wastewater (EG) with PVA-gel beads as biocarrier. Stable chemical oxygen demand (COD) removal efficiencies of 95 % or greater were achieved, and continuous treatment was demonstrated with appropriate parameters being an OLR of 15 kg COD/m^3^/day, NaHCO_3_ added at 400 mg/L, a recycle rate of 15 L/h, and no addition of trace elements addition. A biogas production yield rate of 0.24 m^3^/kg COD was achieved in this study. A large number of long rod-shaped bacteria (*Methanosaeta*), were found with low acetate concentration in the EGSB reactor.

## Background

Ethylene glycol is widely used as a raw material in industrial processes, and many of these processes discharge high-strength ethylene glycol wastewater (EG). Usually a biological process is suggested in treating EG, and good removal performance was achieved with an influent chemical oxygen demand (COD) range between 1000 and 3000 mg/L (Hassania et al. 2014). Despite this, an anaerobic treatment method is preferred due to its simplicity, reduced sludge production and lower power consumption. The formation of microbial granules is a key factor for successful operation of an anaerobic reactor; however, granule formation when treating EG fails to occur (Hulshoff Pol et al. 2004), thus EG treatment plants generally operate with a reduced organic loading rate (OLR).

Polyvinyl alcohol (PVA)-gel beads are thought to be suitable candidate carriers (Wenjie 2008a; Wenjie et al. 2011; Khanh et al. 2011). Wenjie et al. (2009) used cultivated PVA-gel beads to seed a lab-scale anaerobic fluidized bed reactor treating corn steep liquor, and a removal efficiency of 91 % was achieved at an OLR of 27.5 kg-COD/m^3^/day. Wenjie et al. (2011) used PVA-gel beads in an upflow anaerobic sludge blanket (UASB) reactor to treat EG, and successful treatment performance was achieved with addition of sufficient trace elements. The results obtained from the aforementioned studies indicated that PVA-gel beads could display good performance in EG treatment. However, industrial-scale implementation has not occurred due to the lack of understanding of the optimal operational parameters, which are necessary for design.

In this study, PVA-gel beads were used in an expanded granular sludge blanket (EGSB) reactor to evaluate their effectiveness as biocarriers in treating EG. The parameters of EGSB, such as OLR, alkalinity, recycle rate, and trace elements, were investigated for the purpose of further application of this method.

## Methods

### EGSB

**Fig. 1:**
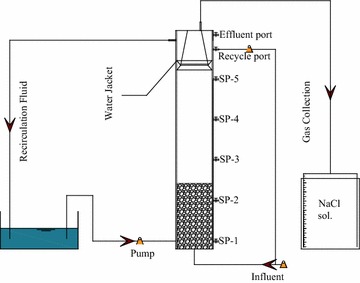
Schematic diagram of EGSB

The EGSB was made of acrylic resin and had a working volume of 3.9 L with a square cross-section of 36 cm^2^ and height of 110 cm. Influent was continuously provided to the EGSB using a peristaltic pump. The reactor had six uniformly located sampling ports and a recirculation pump to adjust the level of bed expansion. The temperature was maintained at 35 °C. Sampling ports were located at heights of 5, 20, 35, 50 and 65 cm above the reactor bottom. A gas–solid separator (GSS) with a simple structure was designed for the collection of generated biogas (Fig. 1). The biogas was separated by the GSS and collected using the method described by Wenjie et al. (2011).

### Inoculation and feeding media

New PVA-gel beads with an average diameter of 3–4 mm were used. At first, in total 2.5 L of digester sludge and 0.95 L of PVA-gel were mixed in one tank with a sequence batch mode. Corn steep liquor (CSL) was used as feed.

After one month, the PVA-gel beads turned yellow in color compared to the white color of unused ones. This means that PVA-gel beads have good biomass affinity. The PVA-gel beads were then separated from the tank and introduced into the EGSB reactor whereby they contributed to about one quarter of the reactor volume. The reactor was started by using EG as influent with an initial COD concentration of about 500 mg/L.

### Analytical methods

COD, suspended solids (SS), volatile suspended solids (VSS), volatile fatty acids (VFAs), alkalinity: Filtered COD (1 µm) were measured by the closed reflux colorimetric method (APHA 1995). SS and VSS in effluent and sludge samples were measured in accordance with Standard Methods (APHA 1995). Alkalinity levels in effluent samples were determined by titration (APHA 1995). VFAs were quantified by using a CTO-10AS liquid chromatograph (Shimadzu, Japan).

Biogas collection and analysis: Biogas was collected through GSS and the volume was measured using an inverted measuring cylinder containing tap water with the pH lowered to 3 using 1 N H_2_SO_4_. Biogas analyses were performed using a GC-14B gas chromatograph (Shimadzu, Japan).

PVA-gel characteristics: the settling velocity of PVA-gel/granules was measured by the method of Wenjie et al. (2008). The amount of sludge (biomass or solids) attached to PVA granules (g VSS/g PVA gel) was determined by the wet weight difference from an average of 30 pairs of new (unused) and granulated PVA-gel.

Scanning electron microscopy (SEM): samples were first washed in a 0.1 M phosphate buffer solution (pH 7.4) for 5 min. The samples were then hardened for 90 min in a 2.5 % glutaraldehyde solution prepared with the buffer solution. Next, the samples were washed in the buffer solution three times for 10 min each and then fixed for 90 min in a 1.0 % OsO4 solution prepared with the buffer solution. After washing the samples three times for 10 min each in the buffer solution, they were dewatered for 10 min each in serially graded solutions of ethanol at concentrations of 10, 30, 50, 70, 90, and 95 %. SEM observations were conducted using a scanning electron microscope (JEOL, JSM-5310LV, Japan).

Methanogenic activity: the methanogenic activity tests were conducted on the PVA-gel beads from the UASB reactor using the method outlined by Wenjie et al. (2011). The tests were performed in one 3.9 L reactor with CSL substrates of equal COD (5 g/L) in order to obtain total methanogenic activity.

## Results and discussion

### Reactor performance

The main operational parameters under various OLRs throughout the study are shown in Table 1. The reactor was started with an OLR of 2.1 kg COD/m^3^/day and a hydraulic retention time (HRT) of 6 h. The influent COD level was increased stepwise to 3500 mg/L (the designed concentration) in 5 steps.

**Table 1:** Summary of conditions used during operation of the EGSB reactor treating EG

Phase	Time (days)	OLR (kg/m^3^/day)	Recycle rate (L/h)	COD_in_ (mg/L)	pH_in_	Trace nutrients (ml/L)	NaHCO_3_ (mg/L)
1	0–14	2.1 ± 0.1	25	523 ± 35	7.56 ± 0.28	7.50	200
2	15–23	4.5 ± 0.1	25	1121 ± 36	7.51 ± 0.24	7.50	400
3	24–35	9.8 ± 0.3	25	2453 ± 64	8.11 ± 0.25	7.50	880
4	36–37	12.0 ± 0.0	25	3004 ± 0	8.12 ± 0.00	7.50	1200
5	38–50	14.6 ± 0.3	25	3640 ± 87	8.02 ± 0.30	7.50	1400
6	51–58	13.4 ± 0.7	20	3360 ± 183	8.22 ± 0.29	7.50	1400
7	59–75	13.2 ± 2.7	20	3304 ± 686	8.16 ± 0.25	3.75	1400
8	76–98	15.2 ± 0.7	15	3807 ± 176	7.84 ± 0.27	3.75	1000
9	99–109	15.0 ± 1.2	15	3743 ± 289	7.62 ± 0.15	3.75	800
10	110–129	14.0 ± 1.0	15	3497 ± 256	7.79 ± 0.21	3.75	600
11	130–149	14.0 ± 0.2	15	3503 ± 46	7.89 ± 0.23	0	600
12	150–168	13.1 ± 1.0	15	3283 ± 261	7.68 ± 0.17	0	400

The reactor was operated continuously for a period of 133 days, during which time the OLR was maintained at 12–15 kg COD/m^3^/day. In phases 6 and 7, the recycle rate was reduced to 20 L/h, and from phase 8, this value was reduced again to 15 L/h. In order to maintain the expanded PVA bed, a recycle rate of 15 L/h must be applied. Thus, no further reduction of recycle rate was carried out in the study. In order to determine the appropriate quantity of added chemicals, the added trace nutrients were reduced to 3.75 ml/L in phase 7 and to 0 in phase 11. In phases 8, 9, 10 and 12, the amounts of NaHCO_3_ added in the influent were 1000, 800, 600, and 400 mg/L, respectively.

**Fig. 2:**
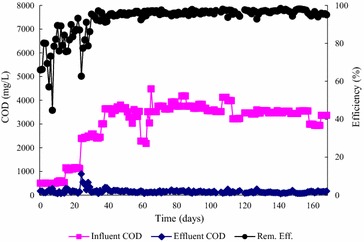
Changes in COD removal for EGSB reactor

The COD removal rate reached a value higher than about 95 % after about 1 month (Fig. 2). The influent COD was then stepwise increased from 500 mg/L to 3500 mg/L in 5 phases. The HRT was maintained at 6 h. After that, the recycle rate, buffer, and nutrients were reduced. There was only a slight change in removal rate as shown in Fig. 2. A stable COD removal rate of about 96 % could be maintained. The EGSB used in this study behaved just like a perfectly mixed reactor in which there is a gas solid separator to retain solids in the reactor. Therefore, reduction of recycle rate from 20 to 15 L/h had no appreciable effect upon reactor performance.

**Fig. 3:**
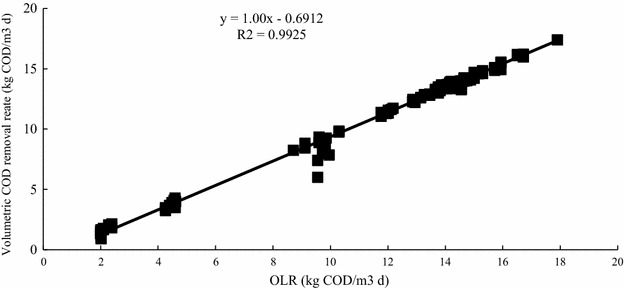
Relationship between OLR and volumetric COD removal rate

The organic loading removal rate was also very good. A value of almost 100 % could be achieved as shown by the linear regression line with a reliable value of about 99 % (Fig. 3). The removal rate decreased slightly when the OLR was less than 10 kg COD/m^3^/day, but soon recovered. This means the proliferation of the bacteria in the EGSB reactor was very fast. Due to the degradability of EG, the concentration had a greater effect on removal rate than HRT.

**Fig. 4:**
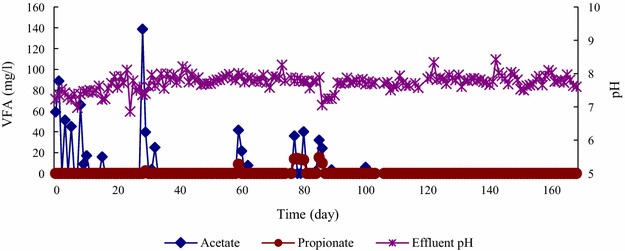
Daily changes in effluent volatile fatty acid (VFA) and effluent pH of the EGSB reactor

The effluent VFA, mainly in the form of acetate, was detected when the influent COD was increased (Fig. 4). Propionate was also occasionally detected but at a concentration below 20 mg/L.

**Fig. 5:**
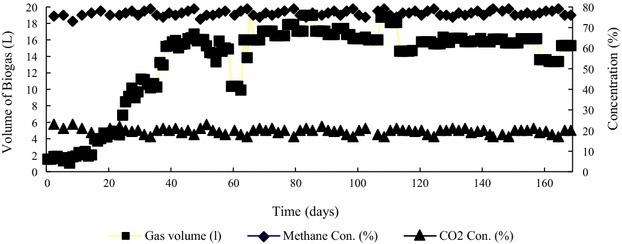
Daily changes in biogas production of EGSB reactor

The biogas production volume was calculated according to the collected biogas in one set time. A biogas production yield rate of 0.24 m^3^/kg COD was achieved in this study (Fig. 5). The CH_4_ and CO_2_ contents were stable during the experiment period.

**Fig. 6:**
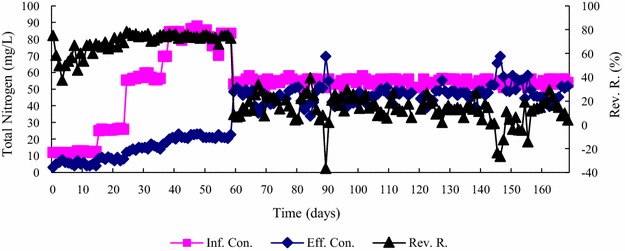
Daily changes in TN removal

**Fig. 7:**
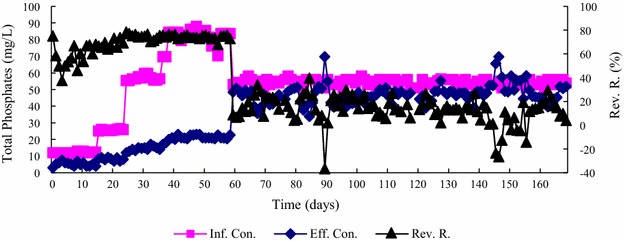
Daily changes in TP removal

Changes in total nitrogen (TN) and total phosphorus (TP) removal are shown in Figs. 6 and 7, respectively. The TN removal efficiency could reach 20 % on average. There was no obvious change even when the amount of NaHCO_3_ added was reduced. On day 59, the trace elements were reduced to 3.75 ml/L. TP removal decreased from 80 to 20 % on average. This indicated that precipitation of orthophosphate might contribute to the TP removal.

### PVA-gel development

**Fig. 8:**
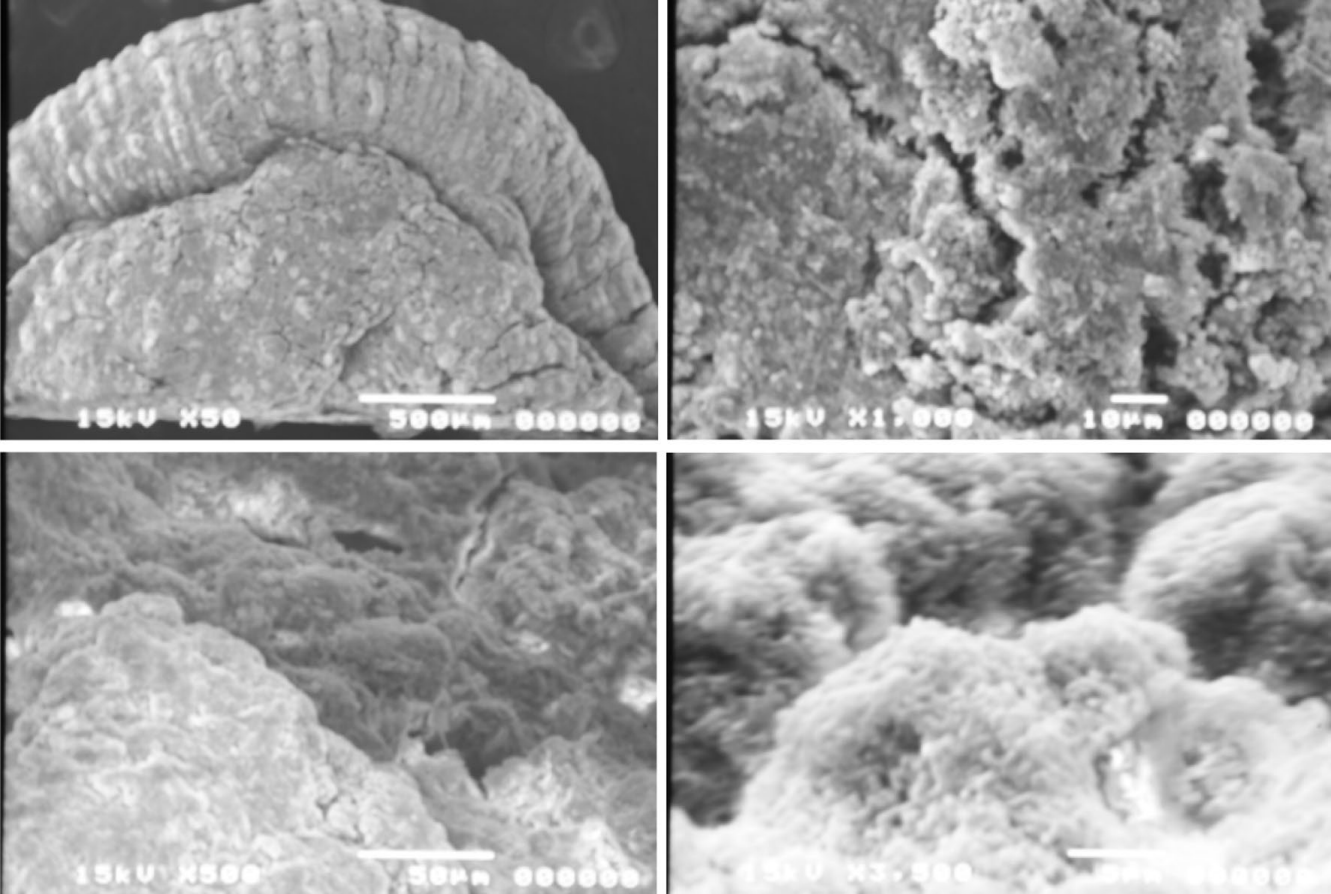
SEM photos of the PVA-gel from EGSB reactor

Figure 8 shows an SEM photo of the PVA-gel obtained in the EGSB reactor. The surface of the PVA-gel beads was covered with dense sludge. A few large cavities were found with small cavities distributed all over the surface for biogas production. The PVA-gel had higher biomass attachment due to the hydraulic conditions of EGSB reactor, which could be proved by the cracks on the surface. Also, the inner part of the PVA-gel beads was observed to be filled with bacteria as shown in Fig. 8.

**Fig. 9:**
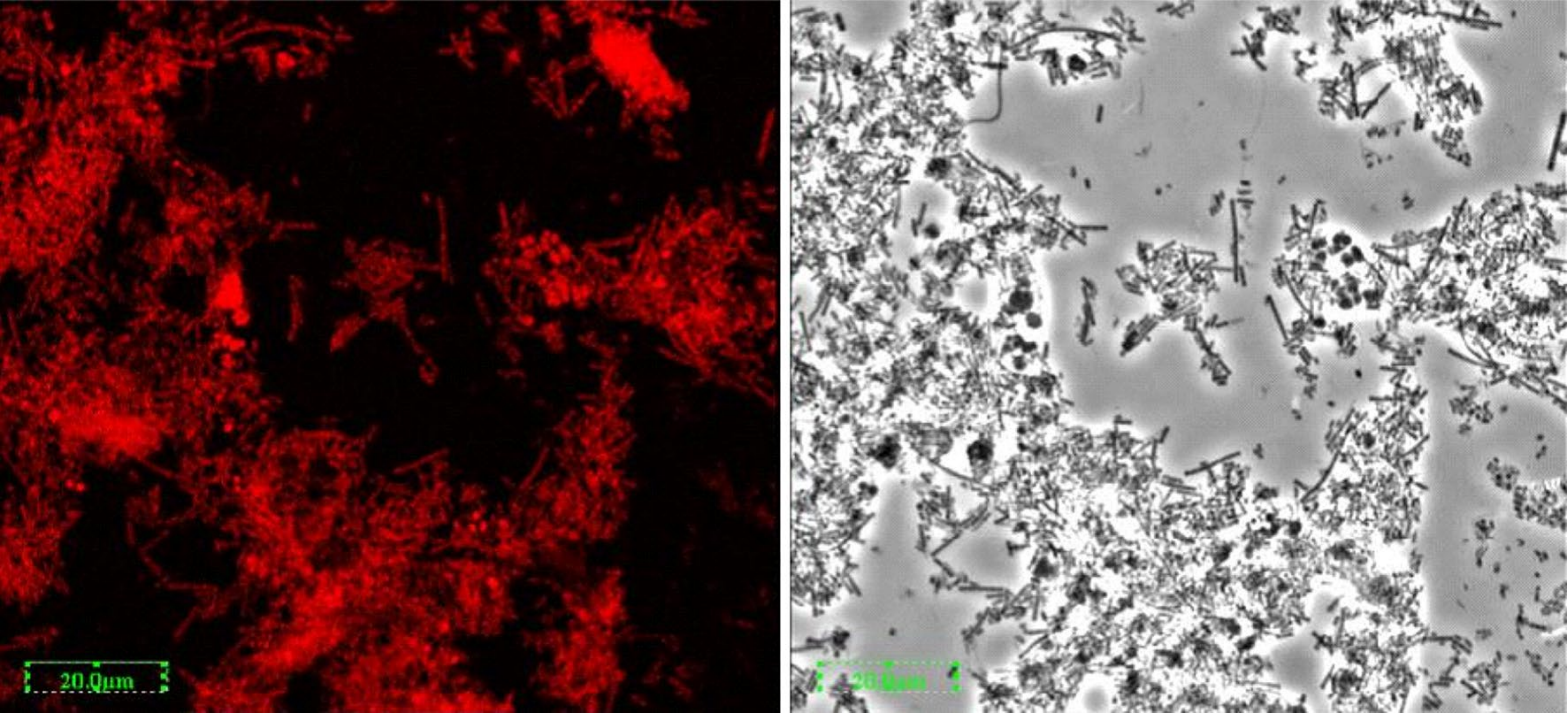
FISH photos of the bacteria attached on the PVA-gel

The bacteria attached to the PVA-gel were composed of a heterogeneous population including short and long rods, cocci and filaments (Fig. 9). The microbial structures on the PVA-gel found in the EGSB reactor were a little different from those in the UASB reactor. A large number of long rod-shaped bacteria (*Methanosaeta*), were found. Low acetate concentration was found in the EGSB reactor, and low acetate concentration was beneficial for long shaped bacteria growth.

**Fig. 10:**
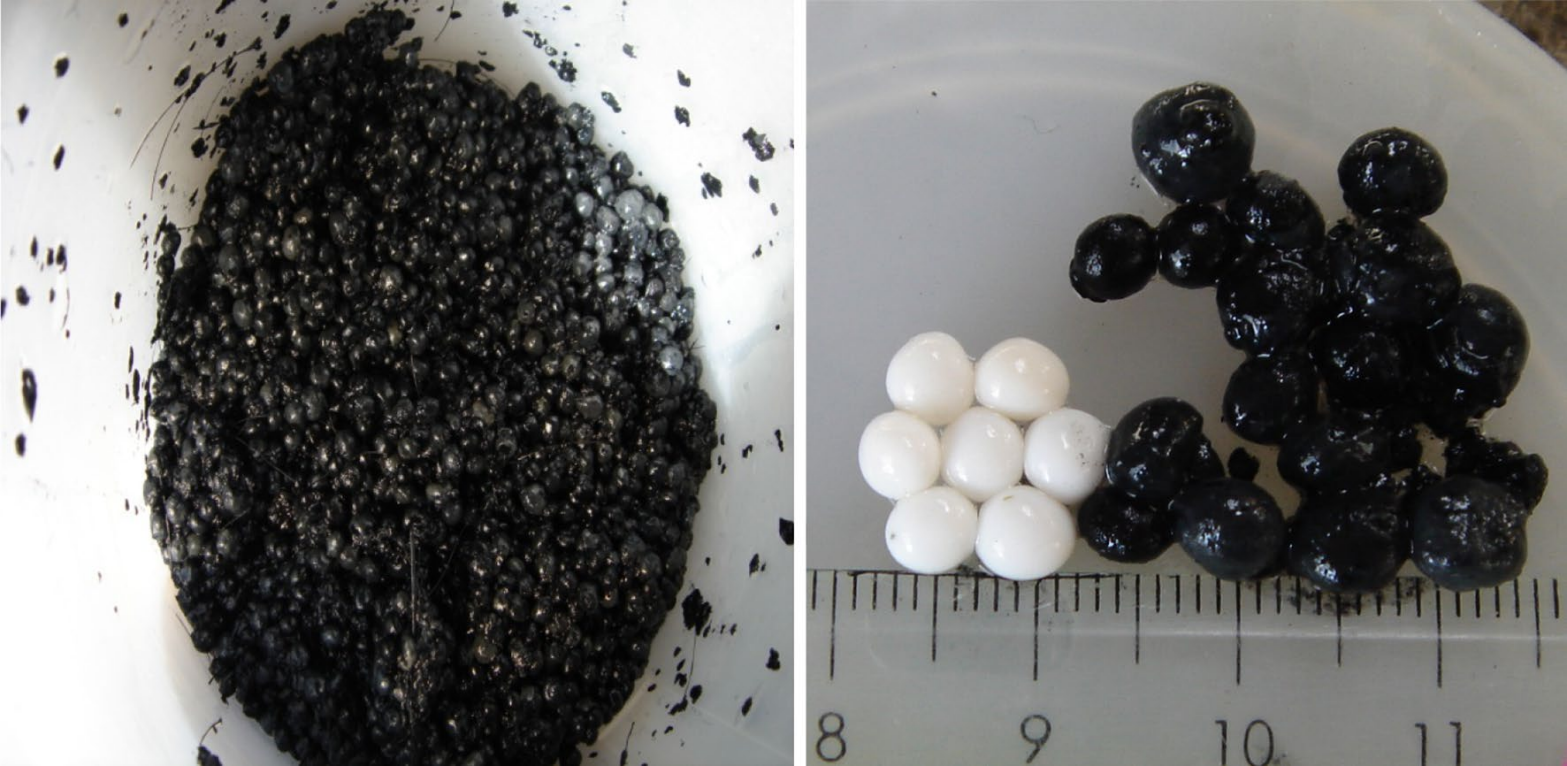
Color changes in PVA-gel of EGSB reactor

At first the PVA-gel beads were white in color. After 1 month of cultivation, the color changed to yellow. At the end of this study, the color of the PVA-gel beads turned completely black (Fig. 10).

The matured black PVA-gel beads had an average settling velocity of 168 m/h (5 cm/s) and 0.83 g VSS/g PVA gel. Compared with the results of the UASB reactor, the settling velocity decreased following the biomass attachment.

The parameters of the UASB and EGSB reactor treating CSL and EG are shown in Table 2. The mixing condition for the EGSB reactor was better than that of the UASB reactor due to the internal recycling. For EGSB reactor, with a higher influent COD, shorter HRT, and higher OLR, a better COD removal efficiency could be achieved. The amount of biomass attached to the PVA-gel increased significantly, especially for treating EG. The specific bacteria in the UASB reactor was mainly composed of *Methanosarcina*, but in the EGSB reactor *Methanosaeta* also contributed to a large part.

**Table 2:** Comparison of UASB and EGSB reactors treating different substrates

Parameter	CSL		EG	
	UASB	EGSB	UASB	EGSB
Mix condition	Bad	Good	Bad	Good
Influent COD (mg/L)	9100	12,900	1500	3283
HRT (h)	8	6	8	6
OLR (kg/m3/day)	22.5	27	11	16
COD removal efficiency (%)	90	92	95	97
Biomass attached (g MLSS/g PVA-gel)	0.93	1.02	0.25	0.83
Specific bacteria	*Methanosarcina*	*Methanosaeta*	*Methanosarcina*	*Methanosaeta*

Compared to the results of the UASB, the cultivated PVA-gel could function well with a shorter HRT and higher OLR due to the higher amount of biomass attached to the PVA-gel beads. Furthermore, the specific bacteria *Methanosarta* was confirmed in the EGSB reactor, which indicated that the mixing condition was better in the EGSB reactor, and the COD level in the reactor was lower.

According to the COD concentration profiles throughout the depth of the reactor, only about 40 mg/l COD was removed from the upper part of the reactor (above 50 cm). It can be concluded that the EG was most degraded by the PVA-gel layer. At the end of the experiment, the concentrations of the added trace nutrients and NaHCO_3_ were 0 and 400 mg/L, respectively. Thus, it can be concluded that the PVA-gel in this study can function effectively as a biocarrier to retain enough sludge in the reactor due to the dilution effect.

## Conclusions

By using PVA-gel cultivated in one batch mode, it was possible to start an EGSB to treat EG. The COD removal efficiency could reach a value higher than 95 % with an OLR of 15 kg COD/m^3^/day. The influent COD was about 3500 mg/L, which was almost the same as real wastewater for this application. Biogas production was about 0.3 m^3^/kg COD with a methane concentration of 77 %. Compared with the results of the UASB reactor, the amount of biomass attached to the PVA-gel in the EGSB reactor was greater. SEM analysis experiment also showed that the inner part of the PVA-gel beads in the EGSB reactor was also filled by bacteria due to the high recycle rate.
